# Using Proper Mean Generation Intervals in Modeling of COVID-19

**DOI:** 10.3389/fpubh.2021.691262

**Published:** 2021-07-05

**Authors:** Xiujuan Tang, Salihu S. Musa, Shi Zhao, Shujiang Mei, Daihai He

**Affiliations:** ^1^Shenzhen Center for Disease Control and Prevention, Shenzhen, China; ^2^Department of Applied Mathematics, The Hong Kong Polytechnic University, Hong Kong, China; ^3^Department of Mathematics, Kano University of Science and Technology, Wudil, Nigeria; ^4^The Jockey Club School of Public Health and Primary Care, Chinese University of Hong Kong, Hong Kong, China; ^5^Shenzhen Research Institute of Chinese University of Hong Kong, Shenzhen, China

**Keywords:** COVID-19, reproduction number, generation interval, latent period, infectious period

## Abstract

In susceptible–exposed–infectious–recovered (SEIR) epidemic models, with the exponentially distributed duration of exposed/infectious statuses, the mean generation interval (GI, time lag between infections of a primary case and its secondary case) equals the mean latent period (LP) plus the mean infectious period (IP). It was widely reported that the GI for COVID-19 is as short as 5 days. However, many works in top journals used longer LP or IP with the sum (i.e., GI), e.g., >7 days. This discrepancy will lead to overestimated basic reproductive number and exaggerated expectation of infection attack rate (AR) and control efficacy. We argue that it is important to use suitable epidemiological parameter values for proper estimation/prediction. Furthermore, we propose an epidemic model to assess the transmission dynamics of COVID-19 for Belgium, Israel, and the United Arab Emirates (UAE). We estimated a time-varying reproductive number [*R*_0_(*t*)] based on the COVID-19 deaths data and we found that Belgium has the highest AR followed by Israel and the UAE.

## Introduction

Emerging and re-emerging infectious diseases pathogens remain an enormous issue for public health and socio-economic growth because they can spread rapidly worldwide. The coronavirus disease 2019 (COVID-19) is a respiratory disease caused by the severe acute respiratory syndrome coronavirus 2 (SARS-CoV-2) (1, 2), and has become a tremendous public health problem affecting every corner of the world (2). Since its appearance in late 2019, about 124 million people contracted and over 2.7 million died worldwide as of March 25, 2021 (2). Until recently, many clinical features and underlying etiology of the SARS-CoV-2 remain unclear. Timely treatment and effective non-pharmaceutical interventions (NPIs) measures against disease are important for effective mitigation (2).

Generation interval (GI), also referred to as the generation time, is the time lag between infection incidents in an infector–infectee pair (3). It is a proxy of serial interval (SI) of infectious disease, which represent the time lag between onsets of the symptoms in an infector–infectee pair (4). The SI and GI are vital biological quantities (epidemic parameters) used for estimating the basic reproductive number (denoted by *R*_0_), which is defined as the number of secondary cases that one infected person will generate on average over the course of his/her infectious period (IP) in a population that is completely susceptible (1, 5), as well as effective reproduction number, *R*_0_(*t*), which determines the average number of secondary cases per infectious case in a population made up of both susceptible and non-susceptible hosts (4). Moreover, the importance of GI is also reflected in the renewal equation $R_{0}\left(t\right)=\frac{I_{t}}{\sum I_{t-k}w_{k}}$, where *w*_*k*_ represents the GI distribution, *I*_*t*_ denotes daily infections, and *R*_0_(*t*) represents the daily instantaneous reproductive number (in this case), which reflects transmission dynamics at a time, *t* (6). Recently, many works have been done to understand and/or estimate the GI, and its proxy, i.e., SI, associated with infectious diseases, including the SARS-CoV and the SARS-CoV-2 (3, 4, 7–13).

Previous reports highlighted that when the SI is larger, the uncertainty and overestimation would be higher (4, 14). The SI (which depends hugely on the incubation period of infectious disease) can be a negative value if the start of symptoms in the infectee happens earlier than the start of the symptoms in the infector (person who transmit the disease) (15–20). The SI can also be a negative value when the incubation period has a relatively wide range than the latent period (LP), which could result in pre-asymptomatic transmission as reported in recent COVID-19 studies (4, 7). However, unlike SI, the GI is solely non-negative according to its definition (10, 21).

The incubation period is the time between infection and the onset of symptoms (21). Although the time of exposure for an individual who transmits the disease (infector) is usually indistinguishable, the time of exposure of an individual who gets the infection (infectee) can be determined by the contact tracing history of the “infector–infectee” pair. This subsequently indicates that for “infector–infectee” pairs, there is a single infector that relates to the infectee epidemiologically. Hence, the incubation periods of infectees can be identifiable. However, the LP differs from the incubation period; it is defined as the time lag between the infection in exposure and onset (beginning) of infectiousness of a typical case (21). Since the beginning of infectiousness is indistinguishable, the LP is unidentifiable. Thus, we noticed that in many diseases (mostly infectious), the mean LP is less than or equal to the mean incubation period (such as COVID-19) (4), whereas some diseases have a long LP, e.g., Ebola virus disease. Note that people infected with Ebola are not infectious until the symptoms started (the incubation period of Ebola varies between 2 and 21 days).

Moreover, the LP is the time interval when an infected individual is unable to transmit the disease, while the time interval during which an infected individual can transmit the disease is called the IP. Both are random variables and are considered independent; thus, the LP and IP are not generally traceable. However, SI is identifiable and well-studied and reported by epidemic models (8, 9). We observed that some studies in the literature did not use the LP and IP appropriately, as the sum of their mean equals the mean GI in susceptible–exposed–infectious–recovered (SEIR)-based models; that is, mean GI = mean LP + mean IP. Using the same notation as in Svensson (3), we have the expectation of the random GI given by *E*(*T*) = *E*(*X*) + *E*(*Y*), where *T* is the random variable representing the GI of the infection, and *X* and *Y* represent the random latent time and random infectious time, respectively. Details on this relation an SEIR-based model can be found in Svensson (3).

Furthermore, it is vital to forecast the size of the outbreak, including infection attack rate (AR), the need for ventilators and hospital beds, the expected severe cases and deaths, the herd immunity threshold, and the vaccine supply needed. All of these are associated with estimate of effective reproductive number, *R*_0_(*t*). Given the important role of *R*_0_(*t*), it is imperative to obtain their estimation more accurately. Therefore, it is crucial to use the proper value for the mean LP and mean IP in SEIR compartmental models.

In the current study, we highlight that using longer IP or LP leads to an overestimation of the reproductive number and other key epidemiological parameters, which, in the initial phase, leads to overestimation of herd immunity threshold and exaggerated control effectiveness. To demonstrate the impact of the GI on *R*_0_(*t*) and to provide more qualitative insights into the use of the GI for controlling infectious disease outbreaks, we fitted a simple model using COVID-19 confirmed death data for Belgium, Israel, and the United Arab Emirates (UAE), by employing a more appropriate LP/IP to reveal and shed light and understanding on the transmission dynamics of COVID-19 in each of these countries. We noticed that Belgium was hit badly by two waves and have high AR than the other two countries. Israel and the UAE have started large-scale vaccination programs.

## Methods

**Figure 1:**
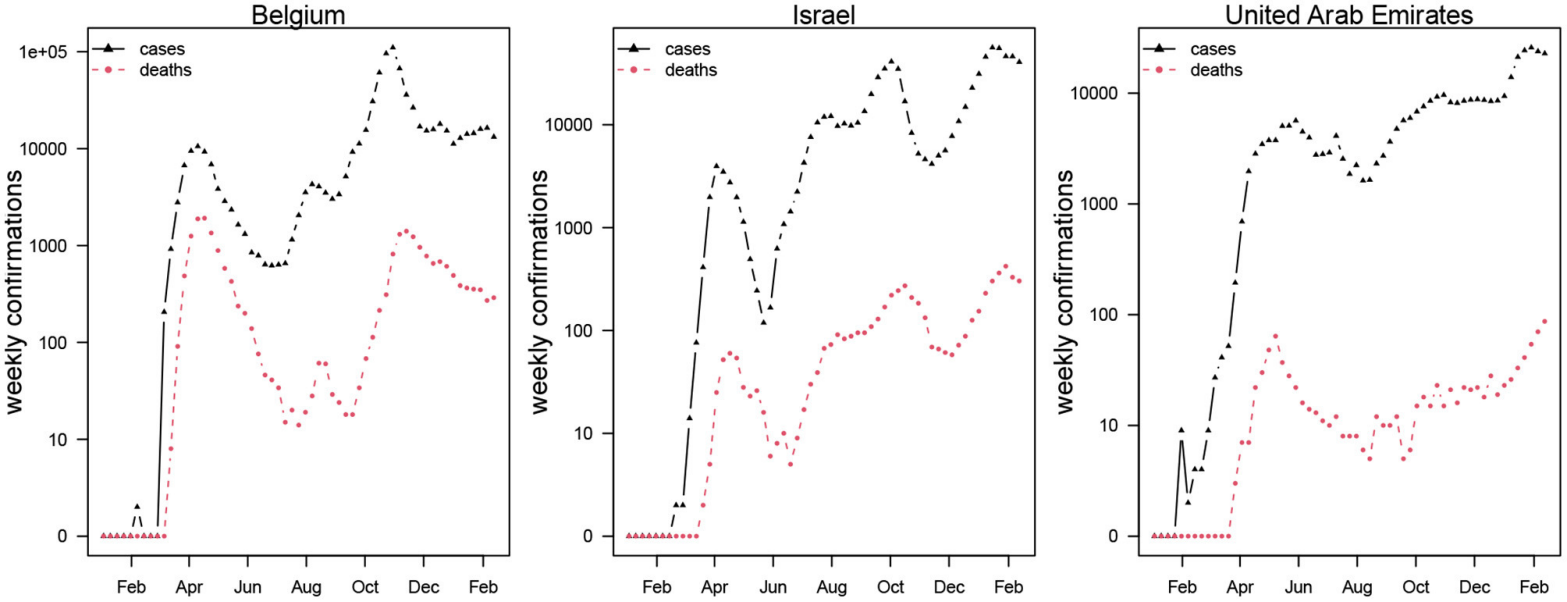
Weekly confirmed cases (in black triangles) and deaths (in red circles) of COVID-19 in Belgium, Israel, and the United Arab Emirates.

This study adopts a SEIHRD model which is widely used in modeling of COVID-19 with minor modification that hospitalization be interpreted as symptomatic cases. We focus on daily reported COVID-19 deaths data retrieved from the official website of the World Health Organization (WHO) public surveillance reports for Belgium, Israel, and the UAE available from https://covid19.who.int/ (2). The time-series distribution of weekly confirmations of COVID-19 cases and deaths in Belgium, Israel, and the UAE is depicted in Figure 1, which shows the patterns of the COVID-19 epidemics in these three countries. The cases and deaths for COVID-19 are represented by black and red dotted curves, respectively. We observed that Israel and the UAE show similar epidemic curve patterns, while Belgium was hit harder with the two waves of COVID-19 outbreaks. The population data for the three countries were obtained from the worldometer, available from https://www.worldometers.info/population/ (22).

Thus, we formulate the following simple epidemic model.

$$\begin{array}{l} Ṡ=-\frac{\beta SI}{N},\\ Ė=\frac{\beta SI}{N}-\sigma E,\\ İ=\sigma E-\gamma I,\\ Ḣ=\theta \gamma I-\kappa H,\\ Ḋ=\theta \kappa H,\\ Ṙ=\left(1-\theta \right)\gamma I+\left(1-\theta \right)\kappa H. \end{array}$$

Here, *S, E, I, R, H*, and *D* represent susceptible, exposed, infection, recovered, hospitalized, and death classes. The parameters β, σ, γ, *and κ* are transmission rate, progression rate from *E* to *I*, recovery rate (for fitting simplicity, we assumed the recovery rate and hospitalization rate to be the same), and the proportion of individuals moving from *H* to *D*, respectively. θ represents both proportions of hospitalization among infection and the proportion of death among hospitalization. Here, we assumed that hospitalization can be interpreted as symptomatic cases. The infection fatality rate equals θ^2^. We fit the daily integrated D to the reported deaths in each country. We assume a negative nominal measurement noise in reporting with an over-dispersion parameter τ. We assume a time-varying β, which is an exponential cubic spline function with the number of nodes as 7, which was evenly distributed over the study period from March 1, 2020, to February 18, 2021. The effective reproductive number is given by *R*_0_(*t*) ≈ β(*t*)/γ.

The model fitting package, POMP, has been widely used in previous studies (23–25). The POMP utilized iterated filtering algorithm, which is based on sequential Monte Carlo (SMC). The method has been extensively validated and used in previous studies. Some recent examples include the work of Stone et al. (26) and He et al. (27). The detailed model-fitting method can be found in many previous studies (23–25).

In the classic susceptible–exposed–recovered-based models, the mean GI of an infectious disease equals the sum of the mean LP and the mean IP (3). The duration of individuals in an exposed/infectious class follows exponential distributions. Due to the discrete time in the simulation of the model, the realized (or simulated) mean LP and mean IP according to He et al. (28) are $LP=\frac{\delta }{1-e^{-\delta \sigma }}$, $IP=\frac{\delta }{1-e^{-\delta \gamma }}$, where δ designates the time discretization step. Thus, σ^−1^ and γ^−1^ are theoretical mean LP and mean IP. The simulated periods were slightly larger than theoretical values due to the time discretization. The discrepancy diminishes when the time step size approaches zero. Hence, the sum of the mean LP and mean IP is estimated at 6.07 days with a 1-day time step size and theoretical 2 days LP and 3 days IP. The mean GI equals 5 days when the time step size approaches zero. Besides σ^−1^ at 2 days and γ^−1^ at 3 days, we set κ^−1^ = 14 days and θ^2^ in the range of 0.5–1% (29). All these petameter values are biologically reasonable.

Therefore, using iterated filtering methods, we fitted a SEIHRD model with an additional death class to reported COVID-19 deaths in the three countries (i.e., Belgium, Israel, and the UAE) to examine the influence of the mean LP and the mean IP for the estimation of reproduction number. We fitted the model to COVID-19 deaths data since COVID-19 mortality data seem less affected by testing policy compared to other diseases.

## Results and Discussion

Based on recently published studies on GI and SI, we observed that the SI (and/or GI) of COVID-19 varies between 5 and 6 days (30). In particular, Ferretti et al. (31) reported the mean GI as 5.0 days, Ganyani et al. (16) used the data for Singapore and Tianjin, China, and found that the mean GI is estimated at 5.20 (3.78–6.78) days and 3.95 (3.01–4.91) days, respectively. In 40 research papers reviewed by Griffin et al. (30) on the GI and SI, about three studies provided an estimate for the mean GI, which varies roughly between 3.95 and 5.20 days. One paper provided an estimate for the median of the GI as 5.0 days (1, 30, 32, 33). Furthermore, Zhang et al. (12) reported that the incubation period of COVID-19 was estimated at 5.2 (95% CI: 1.8–12.4), and the mean IP was estimated at 4.4 (95% CI: 0.0–14.0) from December 24 to January 27, 2020, and 2.6 (95% CI: 0.0–9.0) from January 28 to February 17, 2020.

However, several studies reported the period of disease progression before the infectiousness stage as the LP in an SEIR epidemic model. For example, Yin et al. (34) conducted a modeling study to assess the effectiveness of NPI measures (including contact tracing, facemask wearing, and rapid testing) to curtail the spread of COVID-19 in China. They reported that asymptomatic patients lasted 4.6 days in LP and 9.5 days in IP until removal. See Table 1 for more details. Many studies did not follow the rule that mean GI = mean LP + mean IP <6 days. Therefore, we emphasized that appropriate use of the GI in an epidemiological study is essential to effectively control the COVID-19 outbreaks, because it provides a more accurate estimate on reproduction number for the epidemics, and is crucial for pandemic mitigation planning and forecasting.

**Table 1:** Mean latent period and mean infectious period of COVID-19.

**Mean LP (days)/mean IP (days)**	**Equivalent mean GI (days)**	**References**
None	5.0	(31)
None	5.20 (3.78–6.78) for Singapore 3.95 (3.01–4.91) for Tianjin, China	(16)
5.2 (95% CI: 1.8–12.4) (incubation period)/4.4 (95% CI: 0.0–14.0) from December 24 to January 27, 2020, and 2.6 (95% CI: 0.0–9.0) from January 28 to February 17, 2020	>5.2	(12)
4.6/9.5	14.1	(34)
4.6/5	9.6	(35)
4.3/(5 + 2.1 + 2.9 = 10)	10	(36)
5.1 (incubation period) 12 (95% CI: 2–14)	>12	(37)

**Figure 2:**
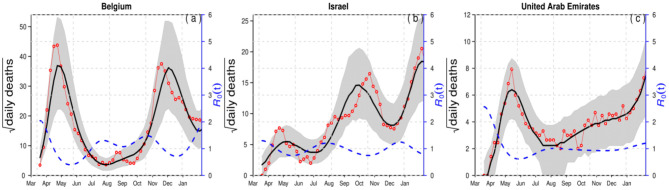
Time-series fitting results of daily confirmed COVID-19 deaths (in red circled) in **(a)** Belgium, **(b)** Israel, and **(c)** the United Arab Emirates represented, respectively. The medium of the simulation is represented by the black curve, and the time-varying effective reproduction number [*R*_0_(*t*)] is denoted in the blue dashed curve. The 95% confidence interval of the simulation is shown by the shaded (gray) region. The mean LP = 2 and the mean IP = 3.

**Figure 3:**
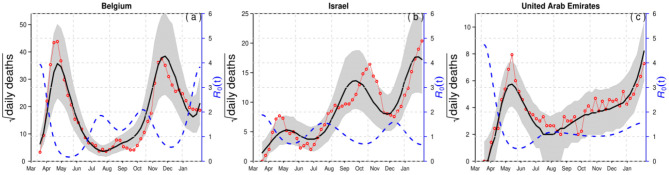
Time-series fitting results of daily confirmed COVID-19 deaths (in red circled) in **(a)** Belgium, **(b)** Israel, and **(c)** the United Arab Emirates represented, respectively. The medium of the simulation is represented by the black curve, and the time-varying effective reproduction number [*R*_0_(*t*)] is denoted in the blue dashed curve. The 95% confidence interval of the simulation is shown by the shaded (gray) region. The mean LP = 3 days and the mean IP = 6 days.

For demonstration purposes, we compared epidemiological dynamics of COVID-19 for some randomly selected countries (Belgium, Israel, and the UAE) while varying LP and IP from 2 and 3 days to 3 and 6 days. We employed the model to the COVID-19 mortality data and obtained the time-series fitting results using the COVID-19 data for Belgium, Israel, and the UAE to quantify the effects of longer GI for the estimation of (time-varying) reproduction numbers. In particular, Figures 2, 3 present the time-series fitting results of the daily confirmed COVID-19 deaths (red circled) with different LP and IP (2 and 3 days to 3 and 6 days) in (a) Belgium, (b) Israel, and (c) the UAE, respectively. The median of the simulation is represented by the black curve, and the time-varying effective reproduction number is denoted by the blue dashed curve. The 95% range of the simulation is shown by the shaded gray region. Based on our results obtained from Figures 2, 3, we discovered that using longer mean LP and mean IP would significantly increase an estimate of reproduction number. Thus, the magnitude of reduction in the initial reproduction number would be much higher in the latter cases (3 and 6 days for LP and IP) than in the proper former cases (2 and 3 days for LP and IP). Besides, a higher initial reproduction number would imply a much higher expected infection AR and herd immunity threshold.

Furthermore, a summary of the results of the COVID-19 infection ARs for Belgium, Israel, and the UAE is presented in Table 2 with reasonable LP and IP values. The choice of LP and IP had an important influence on the estimate of AR, likely due to the choice of the flexible transmission rate in our model and the assumption of the infection fatality rate, which varies between 0.5 and 1%. Appendix Table 1 presents the results of the estimated parameter values including the log likelihood (which is the performance index) for Belgium, Israel, and the UAE. We observed that Belgium has the lowest log likelihood values, indicating that Belgium was hit harder than the other two countries. Also, a summary of the results of the estimated values for the time-varying transmission rate with a fixed number of nodes (denoted by *n*_*m*_) is given in Appendix Table 2. The initial values for the state variables used for the model are given in Appendix Table 3. Therefore, based on the results obtained and the comparison of the epidemic dynamics of COVID-19 for Belgium, Israel, and the UAE with varying LP and IP, we hypothesize that appropriate LP and IP should be used in epidemiological modeling study to effectively mitigate the spread of disease and to provide suitable suggestions of control strategies for public health implementation and policymaking.

**Table 2:** Summary results of the estimated infection attack rates (AR) in Belgium, Israel, and the UAE by February 18, 2021.

**Country**	**Population**	**Death**	**AR**
Belgium	11,589,623	21,041	0.182
Israel	8,655,535	4,634	0.059
UAE	9,890,402	819	0.009

In summary, this study showed that using longer IP or LP leads to overestimation of reproductive number and some other key biological quantities, which, in the initial phase, leads to overestimation of herd immunity threshold and exaggerated control effectiveness. We also quantified the impact of the GI on *R*_0_(*t*) to provide insights into the proper use of GI for controlling infectious disease outbreaks by employing COVID-19 mortality data for Belgium, Israel, and the UAE. We noticed that Belgium was hit badly by two waves and have high AR of 0.182 followed by Israel with AR of 0.059 and the UAE with AR of 0.009, whereas Israel and the UAE have started large-scale vaccination programs. Our proposed epidemic model of the COVID-19 presented in this work is similar to previous models discussed in various studies (3, 8, 23, 25, 27, 38–41), with the assumption that hospitalization is considered as symptomatic cases. We employed COVID-19 mortality data for Belgium, Israel, and the UAE in the model to demonstrate the impact of the GI on the reproductive number, and to provide more qualitative insights into the use of the LP/IP for modeling infectious diseases. For future work, we plan to extend our paper by designing a technique that would be used to test the reliability and efficiency of proper validation and performance indices in relation to our fitting results as well as to adapt our existing technique for the design and analysis of the complex scenario. Furthermore, we plan to integrate the existing technique for system implementation modeling and to come up with a model protocol to check and test the reliability of the model and its futures on disease dynamics for timely and effectual control and prevention.

## Conclusions

Mean LP, IP, and GI are essential quantities in epidemiological modeling studies that are used for estimation of reproductive number of an infectious disease. For COVID-19, current knowledge showed that the mean GI (mean LP + mean IP) varies between 5 and 6 days, which implies that the mean LP and IP in SEIR models should be around 2–3 days, respectively. We emphasized that this estimate should be used to provide a more reasonable estimation of reproductive number (*R*_0_) and other key epidemic quantities, which helps in providing suggestion to policymakers to curtail the spread of an infectious disease. We showed that the estimated *R*_0_ (*t*) for Belgium, Israel, and the UAE are elevated substantially when longer LP and IP are used. Since now vaccination programs are ongoing in these countries, all modeling fitting is timely to lay the groundwork for the efficacy evaluation of the vaccination programs in these countries.

## Data Availability Statement

The original contributions presented in the study are included in the article/Supplementary Material, further inquiries can be directed to the corresponding author/s.

## Author Contributions

All authors listed have equally contributed to the work and approved it for publication.

## Conflict of Interest

DH was supported by an Alibaba China Co. Ltd. Collaborative Research grant (ZG9Z). The remaining authors declare that the research was conducted in the absence of any commercial or financial relationships that could be construed as a potential conflict of interest.
