# Supplementary material for: Using Proper Mean Generation Intervals in Modeling of COVID-19
Source: Front Public Health. 2021 Jul 5;9:691262. doi: 10.3389/fpubh.2021.691262 (PMC8287506; doi:10.3389/fpubh.2021.691262)
Supplement: Supplementary file 1 [file Table_1.pdf]

Appendices

Title: Using proper mean generation intervals in modelling of COVID-19.

File name: Supplementary material 1 - Appendix.docx

Title of data: Appendix

Description of data: Contains the following.

Appendix Table 1. Summary table of estimation results of the parameters (using log likelihood).

Appendix Table 1. Summary table of estimation results of the time varying transmission rate with number of nodes ($n_m = 7$) fixed.

Appendix Table 2. Summary table for initial values of state variable of the model.

Appendix Table 1*:* Summary table of estimation results of the parameters (using log likelihood).

Country	loglik	loglik.sd	σ	θ	γ	κ	Pop size
Belgium	-284.8281	0.0229	182.5	0.1	121.6667	26.0714	11589623
Israel	-229.7767	0.034	182.5	0.0954	121.6667	26.0714	8655535
UAE	-139.7897	0.0829	182.5	0.0933	121.6667	26.0714	9890402
Belgium	-285.0237	0.026	121.6667	0.0935	60.8333	26.0714	11589623
Israel	-230.729	0.039	121.6667	0.1	60.8333	26.0714	8655535
UAE	-138.8795	0.0593	121.6667	0.0826	60.8333	26.0714	9890402

***Appendix Table 2**:* Summary table of estimation results of the time varying transmission rate with number of nodes ($n_m = 7$) fixed.

log.beta	log.beta1	log.beta2	log.beta3	log.beta4	log.beta5	log.beta6
5.5159	3.9121	4.9633	4.8712	5.1678	4.4799	5.5484
5.0615	4.5097	4.9234	4.8167	4.5318	5.0215	4.5114
5.7429	4.4287	4.6888	4.7855	4.7286	4.7858	4.9997
5.4773	2.3899	4.5058	4.3241	4.797	3.5729	5.4488
4.7398	3.7754	4.4478	4.2501	3.7927	4.5705	3.7169
5.6623	3.6152	4.0472	4.2476	4.1243	4.2418	4.5581

***Appendix Table 3**:* Summary table for initial values of state variable of the model.

Tau	S.0	E.0	I.0	T.0	D.0	R.0
0.1702	11010142	4547	4547	455	45	569887
0.1286	8222758	392	392	39	4	431950
0.0016	9395882	11	11	1	0	494496
0.1521	11010142	7231	7231	723	72	564225
0.1243	8222758	471	471	47	5	431783
0.0016	9395882	22	22	2	0	494474
